# Evaluating the feasibility of Cas9 overexpression in 3T3-L1 cells for generation of genetic knock-out adipocyte cell lines

**DOI:** 10.1080/21623945.2021.1990480

**Published:** 2021-12-16

**Authors:** Tomás Suchý, Isabell Kaczmarek, Tomislav Maricic, Christian Zieschang, Torsten Schöneberg, Doreen Thor, Ines Liebscher

**Affiliations:** a Department of Molecular Biochemistry, Rudolf Schönheimer Institute of Biochemistry, Medical Faculty, Leipzig University, Leipzig, Germany; b Department of Evolutionary Genetics, Max-Planck-Institute for Evolutionary Anthropology, Leipzig, Germany

**Keywords:** Adipocytes, Cas9, CRISPR/Cas, adipocyte function, 3T3-L1, genetic knock-out

## Abstract

Cell lines recapitulating physiological processes can represent alternatives to animal or human studies. The 3T3-L1 cell line is used to mimic adipocyte function and differentiation. Since transfection of 3T3-L1 cells is difficult, we used a modified 3T3-L1 cell line overexpressing Cas9 for a straightforward generation of gene knock-outs. As an example, we intended to generate 3T3-L1 cell lines deficient for adhesion G protein-coupled receptors *Gpr64/Adgr2* and *Gpr126/Adgr6* using the CRISPR/Cas approach. Surprisingly, all the generated knock-out as well as scramble control cell lines were unresponsive to isoprenaline in respect to adiponectin secretion and lipolysis in contrast to the wild type 3T3-L1 cells. We, therefore, analysed the properties of these stable Cas9-overexpressing 3T3-L1 cells. We demonstrate that this commercially available cell line exhibits dysfunction in cAMP signalling pathways as well as reduced insulin sensitivity independent of gRNA transfection. We tried transient transfection of plasmids harbouring Cas9 as well as direct introduction of the Cas9 protein as alternate approaches to the stable expression of this enzyme. We find that transfection of the Cas9 protein is not only feasible but also does not impair adipogenesis and, therefore, represents a preferable alternative to achieve genetic knock-out.

## Introduction

To reduce the number of animal studies *in vitro* cell and tissue models can provide an alternative to mimic physiological tissues and their functional processes [1]. Furthermore, experiments with cell lines are less time-consuming, show higher reproducibility, and genetic manipulation is easier to achieve. To analyse the impact of specific genes or proteins on cellular function, overexpression or knock-down (KD) approaches based on transcript interference are commonly used. However, many cell lines are hard to transfect with standard methods and the low stability and efficiency of e.g. siRNA may further reduce KD efficiency. Thus, gene-editing tools have been established to overcome these problems. Among them the CRISPR/Cas9-mediated techniques have gained large popularity [2].

The 3T3-L1 cell line is one of the most widely used cell culture model for adipocyte function and differentiation [3]. This mouse fibroblast-derived preadipocyte cell line was established in the 1970s [4]. Unfortunately, transfection of these cells, especially of differentiated adipocytes, shows low efficiency [5]. Therefore, overexpression of genes is often impossible and siRNA-mediated gene KD has been mainly limited to preadipocytes [6–8]. A targeted genome editing approach using the CRISPR/Cas9 system represents a superior method for efficiently generating transgenic 3T3-L1 cells, which stably overexpress or knock-out (KO) the genes of interest while the introduction of inducible promoters allows for a timely expression modulation [9]. Because the low transfection efficiency of this cell-line limits also the transient expression of the Cas9 gene, a stably Cas9-expressing 3T3-L1 cell line may provide some advantages to achieve sufficient genome-editing. Such cell lines have already been successfully generated for 3T3-L1 [10,11] and mesenchymal [12] cells to study gene KO in adipocytes. Furthermore, Cas9-expressing 3T3-L1 cells are commercially available from different distributers.

To analyse the impact of receptor loss, we generated KO cell lines for the receptors *Gpr64/Adgrg2* and *Gpr126/Adgrg6* as well as a scramble control cell line, which was transfected with a scrambled guide RNA. Both receptors belong to the large superfamily of G protein-coupled receptors (GPCRs) and are grouped into the Adhesion GPCR (aGPCR) family [13–15]. For both receptors, coupling to G proteins has been demonstrated [16–21] and it has been shown that they are functionally relevant in adipocytes [22]. However, previous experiments in 3T3-L1 cells were conducted using an siRNA-based approach yielding an unstable KD that did not last over the whole course of differentiation. We aimed to overcome these limitations with CRISPR/Cas9-generated receptor KOs. Using these novel cell lines, we observed impaired adipogenesis in 126ko and 64ko cells with significantly reduced expression of the adipogenesis markers *Pparγ* and *Cebpα*, as one would expect from the siRNA-mediated KD [22]. Further experiments regarding adipocyte function revealed an unexpectedly low response to stimuli like isoprenaline as well as insulin. We, therefore, investigated basic parameters of adipogenesis and adipocyte function comparing wild-type 3T3-L1 cells (wt) to non-transfected Cas9-overexpressing cells (Cas9). Here, we show that already Cas9 overexpression has a considerable effect on adipogenesis, which appears to be mediated by a significantly reduced response to differentiation signals mediated by G protein-dependent pathways. Alternate approaches of introducing Cas9 into 3T3-L1 cells were specifically successful when the Cas9 protein was directly transfected into the cells, which proves that Cas9-mediated gene KO is feasible but has to be correctly controlled.

## Results

### Establishing GPR126 and GPR64 KO cell lines

**Figure 1.**
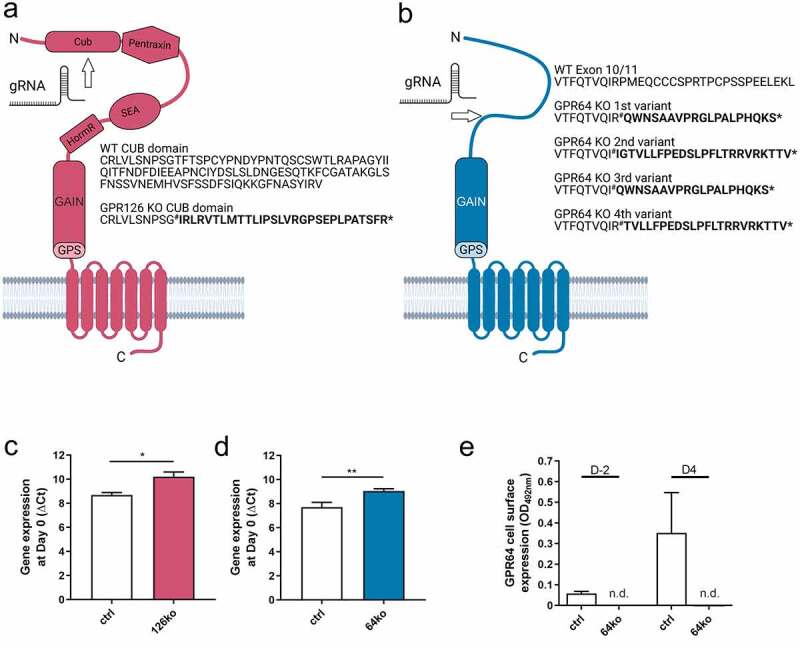
Generation of *Gpr126* and *Gpr64* knock-out cell lines. (a, b) 3T3-L1 cells constitutively expressing the Cas9 protein were transfected with gRNA targeting *Gpr126* (a) or *Gpr64* (b). The targeted protein regions are shown schematically; the amino acid sequence displays the location of the Indel (#) and the following stop codon (*), as well as the amino acids differing from wt (bold); GPS: GPCR proteolytic site; GAIN: GPCR autoproteolysis-inducing domain; HormR: Hormone Receptor domain; SEA: Sea urchin sperm protein, Enterokinase, Agrin; CUB: Complement C1r/C1s, Uegf, Bmp1. (c, d) Gene expression of *Gpr126* (C, n = 3) and *Gpr64* (D, n = 5) (normalized to *Actb2*, Ct: 15.28 ± 0.214) is shown in preadipocytes of the given cell lines. (e) GPR64 cell surface expression was determined in ctrl and 64ko cells in preadipocytes and during differentiation at day 4. Data are shown as OD_492nm_ readings after detracting background (OD_492nm_: 0.04 ± 0.02). Given is the mean ± SEM (n = 5). Significance was determined by paired t-test (*p < 0.05; **p < 0.01). Figure is created by using Biorender.com

We previously demonstrated that *Gpr64* and *Gpr126* influence adipocyte function and adipogenesis [22]. However, as siRNA-mediated KD of these receptors was neither complete nor stable until the final days of differentiation, we generated *Gpr64* and *Gpr126* knock-out cell lines using the CRISPR/Cas9 approach. Thus, we used a commercially available 3T3-L1 cell line constitutively expressing the Cas9 gene. Guide RNA (gRNA) was designed to target the N-terminus of each receptor (Figure 1a, b). Sequencing analysis revealed deletions leading to premature stop codons (Figure 1a, b, Suppl. Figure S1) indicating a functional receptor knock-out in both cell lines.

To evaluate the success of this approach, mRNA levels of the given receptor were compared between cells transfected with scrambled control (ctrl) and receptor-specific guide RNA (126ko or 64ko). We observed significant transcript reduction for both receptors in the respective knock-out cell line compared to ctrl cells, most-probably due to non-sense mediated decay (Figure 1c, d). GPR64 cell surface expression was absent in 64ko cells at any investigated time point during differentiation (Figure 1e). Unfortunately, there is no antibody available targeting GPR126 to evaluate the loss of expression, thus protein levels were not examined for this receptor.

### Adipocyte differentiation is altered in 126ko and 64ko cells

**Figure 2.**
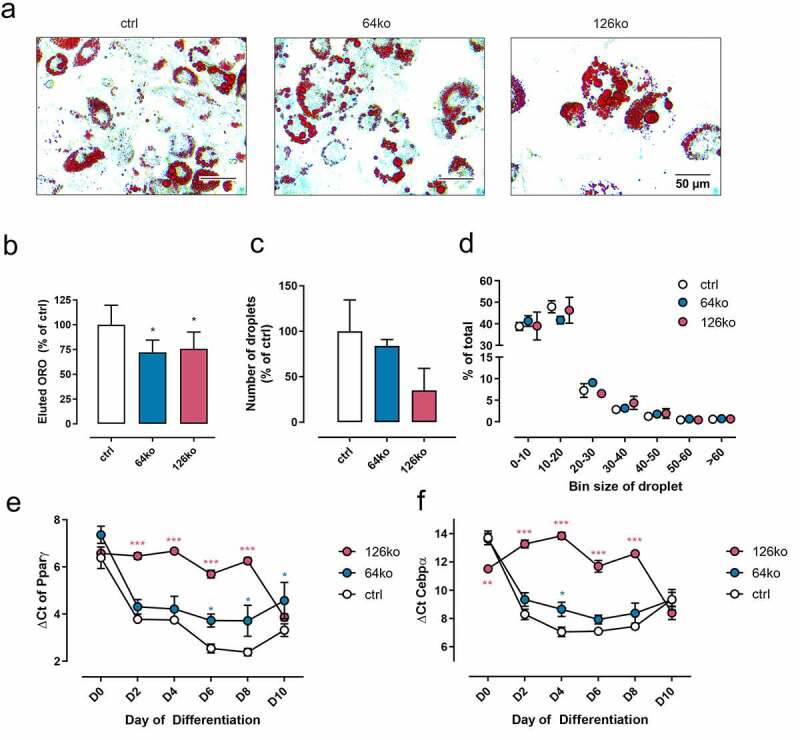
The effect of *Gpr64* and *Gpr126* knock-out on adipogenesis in 3T3-L1 cells. Differentiation was analysed in ctrl, 64ko and 126ko adipocytes. (a) 3T3-L1 mature adipocytes stained with ORO (D10). (b) Total lipid accumulation was measured by eluted ORO in mature adipocytes (n = 8). (c) Lipid droplet number was counted in a defined area (0.2664 mm^2^) in mature adipocytes (n = 3). (d) Additionally, the size distribution of lipid droplets was measured and categorized (n = 3). (e, d) The expression of differentiation markers *Pparγ* (e) and *Cebpα* (f), normalized against *Actb2* (Ct = 16.62 ± 0.282), was measured every other day of differentiation (n = 5). Given is the mean ± SEM. Significance was determined using paired one-way ANOVA compared to ctrl (*p < 0.05; **p < 0.01; ***p < 0.001)

In a next step, we analysed the morphology of mature 64ko and 126ko adipocytes in comparison to ctrl cells. After 10 days of differentiation, which usually yields mature adipocytes, cells were stained with ORO to analyse lipid content. We observed a reduced number of differentiated cells in 64ko and, even more pronounced, in 126ko cells compared to ctrl (Figure 2a), which was confirmed by a significantly reduced amount of eluted ORO (Figure 2b). Furthermore, we found a trend towards a lower number of lipid droplets in 126ko cells (Figure 2c), whereas the distribution of droplet size was not affected (Figure 2d). The loss of *Gpr64* alters lipid accumulation during adipocyte differentiation to a much lesser extent than loss of *Gpr126* (Figure 2a-c), which is in line with previous observations using siRNA transfection [22].

A reduced lipid accumulation and droplet number are usually an indicator of impaired adipocyte differentiation. Thus, we examined the expression of established markers for differentiation, *Pparγ* and *Cebpα* [23,24] (Figure 2e, f). Both markers were significantly lower expressed in 126ko cells from day 2 to day 8 of the differentiation process. These days represent the important days of differentiation; while from day 8 on, the cells mainly increase their size through lipid accumulation [25]. In 64ko cells, changes in *Pparγ* and *Cebpα* transcript levels were significant at days 6 to 10 for *Pparγ* and day 4 for *Cebpα*, again indicating a slighter interference of *Gpr64* KO with adipogenesis compared to KO of *Gpr126* (Figure 2e, f).

### Analysing the sensitivity to external stimuli connected to adipocyte metabolism

**Figure 3.**
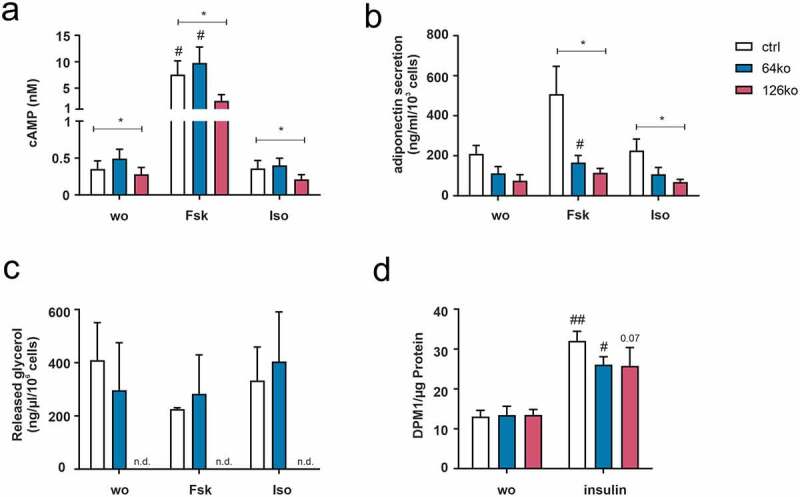
Analysis of cAMP- or insulin-mediated adipocyte functions in ctrl, 64ko and 126ko cells. (a) cAMP accumulation was measured in preadipocytes after stimulation with forskolin (Fsk) and isoprenaline (Iso) (n = 5). (b, c) Secreted adiponectin (n = 5) (b) and released glycerol (n = 3) (c) were analysed in the supernatant of mature adipocytes after forskolin (Fsk) and isoprenaline (Iso) stimulation (n.d. not determinable). (d) Glucose uptake was measured in mature adipocytes (n = 3) after stimulation with insulin. Data is shown as mean ± SEM. Statistical significance was identified by paired one-way ANOVA. Black asterisks show significance compared to ctrl cells at the given condition, black hashes show significance for each cell line compared to without stimulation condition (wo) (*/# p < 0.05; **/## p < 0.01)

One major pathway activated by GPCRs including GPR64 and GPR126 is the change of intracellular cAMP levels either by activation of G_s_- or G_i_-proteins. As cAMP is mandatory for induction of preadipocyte differentiation, GPCRs activating or inhibiting adenylyl cyclase are involved in the regulation of adipogenesis [26]. Therefore, we initially tested the capability of all three cell lines (ctrl, 64ko, 126ko) to respond to external stimuli initiating cAMP production. Thus, cells were stimulated with either forskolin, an unspecific activator of adenylyl cyclases, or isoprenaline, an agonist of β-adrenergic receptors, which are both well known for their importance in adipocytes [27]. Interestingly, 126ko cells displayed already reduced cAMP levels under basal conditions compared to ctrl cells (Figure 3a). Furthermore, forskolin initiated an increase in intracellular cAMP in all cell lines compared to unstimulated conditions; however, in 126ko cells the increase is significantly reduced compared to ctrl cells (Figure 3a). Surprisingly, isoprenaline was not able to induce an increase in intracellular cAMP in any of these cell lines (Figure 3a).

As deficiency in regulating cAMP levels is not only connected to impairment in adipogenesis, we also tested adipocyte function connected to this second messenger. Thus, we analysed secretion of adiponectin after stimulation with forskolin and isoprenaline (Figure 3b). Again, forskolin and isoprenaline did not induce an appropriate response in all three cell lines even though these effects have been previously demonstrated in 3T3-L1 cells [22,28]. Furthermore, neither forskolin nor isoprenaline were capable of inducing lipolysis in ctrl and 64ko cells, whereas no lipolysis was detected in 126ko cells (Figure 3c) most probably due to majorly impaired differentiation (see Figure 2). These data indicate that general cAMP-dependent mechanisms in adipocyte biology are negatively affected in all three cell lines tested.

To analyse if these cell lines are still sensitive to insulin, we measured glucose uptake, which was significantly increased in ctrl and 64ko cell lines under insulin stimulation (Figure 3d), indicating that knock-out of these receptors did not essentially interfere with insulin-mediated function.

Overall, all data indicate an alteration of cAMP-mediated adipocyte function already present in ctrl cells, which cannot be caused by the knock-out of the targeted Gs protein-coupled receptors GPR64 and GPR126.

### Functional analysis of 3T3-L1 wt and Cas9 cells

**Figure 4.**
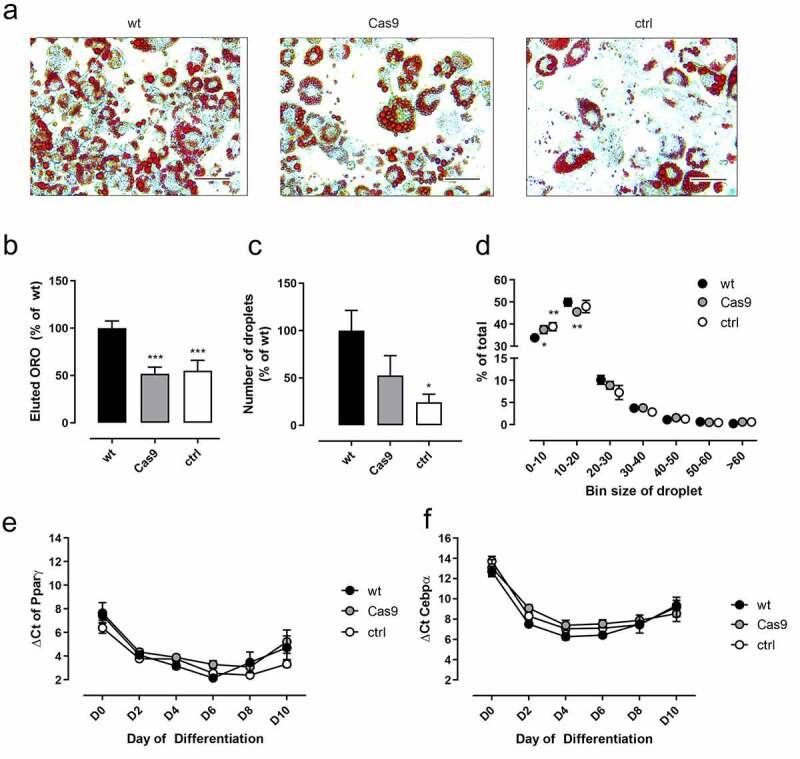
Influence of transfection and constitutive Cas9 expression on adipogenesis. Differentiation was analysed in wt, Cas9, and ctrl cells. (a) Mature 3T3-L1 adipocytes (D10) after ORO staining. (b) Total lipid accumulation was measured by eluted ORO in mature adipocytes (n = 8). (c) In mature adipocytes the number of lipid droplets was determined in a defined area (0.2664 mm^2^) (n = 3). (d) Additionally, the size distribution of lipid droplets was measured (n = 3). (e, f) The expression of differentiation markers *Pparγ* (e) and *Cebpα* (f), was measured during differentiation and normalized against *Actb2* (Ct = 16.62 ± 0.282) (n = 5). Given is the mean ± SEM. Significance was determined using a paired one-way ANOVA compared to wt; *p < 0.05; **p < 0.01; ***p < 0.001

To decipher whether the defects seen in ctrl cells were due to the transfection of gRNAs or if the overexpression of Cas9 already causes this phenotype, we compared adipogenesis of conventional (wt), Cas9 overexpressing (Cas9), and scramble control gRNA-transfected Cas9 overexpressing (ctrl) 3T3-L1 cells (Figure 4). We observed reduced differentiation in Cas9 and ctrl cells compared to wt (Figure 4a), which was also seen in significantly reduced ORO staining and a reduced number of droplets in these cells (Figure 4b, c). Detailed analysis of droplet size showed a significant shift towards the small droplet bin size in Cas9 and ctrl cells (Figure 4d). Surprisingly, these changes were not caused by significant changes in *Pparγ* and *Cebpα* expression (Figure 4e).

**Figure 5.**
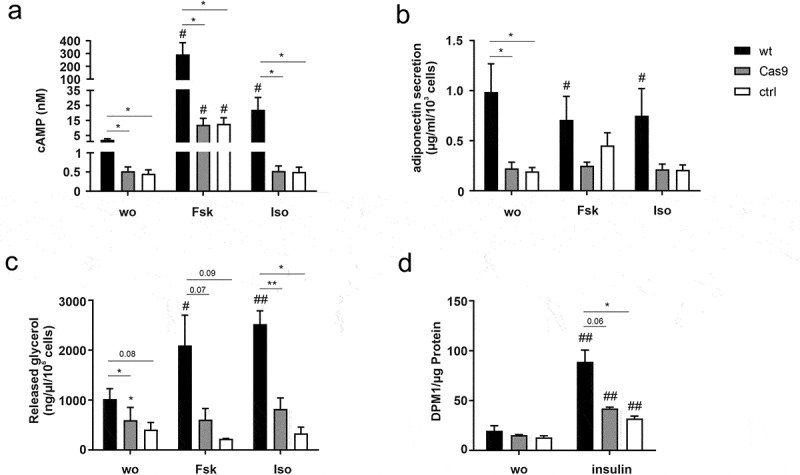
Impairment of adipocyte functions due to Cas9 overexpression. (a) Cyclic AMP accumulation was analysed in 3T3-L1 preadipocytes of wt, Cas9, and ctrl cells on a basal level and after stimulation with forskolin (Fsk) and isoprenaline (Iso) (n = 7). (b) Mature adipocytes were analysed for adiponectin secretion (n = 6). (c) Released glycerol after Fsk and Iso stimulation was determined as a measure of lipolysis (n = 3). (d) Glucose uptake was measured with and without insulin stimulation on differentiated cells (n = 3). Data is shown as mean ± SEM. Statistical significance was identified by paired one-way ANOVA. Black asterisks show significance compared to wt cell line, black hashes show significance compared to no stimulation (wo) (*/# p < 0.05, **/## p < 0.01)

Analysis of the effects of forskolin and isoprenaline stimulation on adipocytes demonstrated that the stable expression of Cas9 already caused significant interference with cAMP signalling and associated functions (Figure 5). Forskolin induced a significant increase in cAMP formation, yet, this effect was reduced in Cas9 and ctrl cells compared to wt. Basal cAMP levels were reduced in Cas9 and ctrl cells and could not be elevated through isoprenaline (Figure 5a), as it has been observed in 64ko and 126ko cells (see Figure 3a). Consequently, forskolin and isoprenaline were neither able to induce a reduction in adiponectin secretion (Figure 5b) nor an increase in lipolysis (Figure 5c) in Cas9 and ctrl cells whereas wt cells showed a significant signal in all assays.

Although insulin was again able to induce glucose uptake in all three investigated cell lines, this effect was significantly lower in Cas9 and ctrl cells indicating that cell lines derived from Cas9 cells are also altered in insulin signalling pathway (Figure 5d).

### Gene expression analysis of wt, Cas9, and ctrl cells reveals significant differences

**Figure 6.**
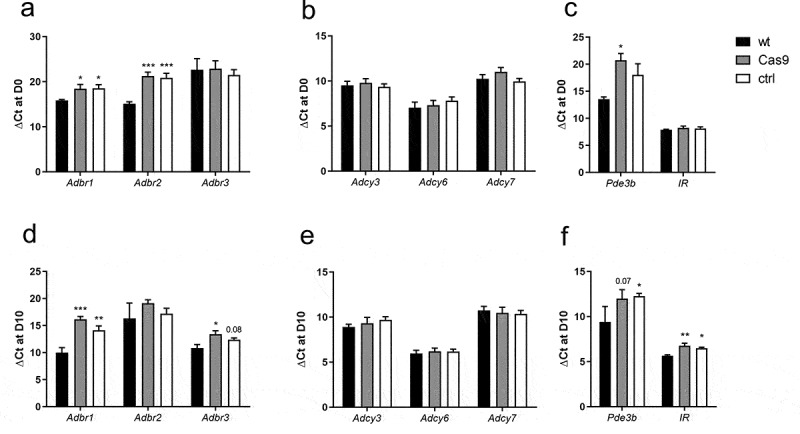
mRNA-expression for genes involved in β-adrenergic signalling, cAMP-accumulation and -degradation as well as insulin signalling in preadipocytes (a-c) and mature adipocytes (d-e). (a, d) Expression of β adrenergic receptors was analysed as isoprenaline stimulation did not induce cAMP accumulation in Cas9-derived cell lines. (b, e) As forskolin stimulation led to significantly lower cAMP values, expression of adenylyl cyclases was monitored. (c, f) Cas9 overexpression led to impaired insulin-mediated glucose uptake, we therefore analysed *IR* and *Pde3b* expression. Ct values of the given gene are normalized to the corresponding *Actb2* values (Ct = 15.29 ± 0.230). The data is shown as mean ± SEM (n = 4–7). Statistical significance was identified using one-way ANOVA. Black asterisks show significance compared to wt (* p < 0.05; ** p < 0.01; *** p < 0.001)

As stable Cas9 overexpression already leads to a diminished cAMP accumulation and an insensitivity towards isoprenaline in 3T3-L1 preadipocytes, we monitored expression of β-adrenergic receptors (*Adbr*). We found significantly lower expression of *Adbr1* and *Adbr2* in Cas9 and ctrl cells whereas expression of *Adbr3* was not different from wt cells (Figure 6a). Thus, the reduced isoprenaline sensitivity was most probably influenced by decreased *Adbr1* and *Adbr2* expression. Due to a lower cAMP accumulation after forskolin stimulation, we determined the mRNA expression levels of adenylyl cyclases (*Adcy) 3, 6*, and *7*. However, no changes in expression were found (Figure 6b). Expression of *Adcy8* was analysed but not detectable in 3T3-L1 cells. Regarding the remaining adenylyl cyclases, either expression was not reported in adipose tissue (*Adcy1, 2, 10*), or specific primers for qPCR analyses could not be designed (*Adcy4, 5, 9*) due to non-specific amplification or a low doubling efficiency per cycle. Because the reduced basal cAMP as well as cAMP accumulation (Figure 3) cannot be attributed to adenylyl cyclase expression, we suspected phosphodiesterases (*Pde*) to play a role in these findings. We analysed expression of *Pde3a*, which was only detectable in traces, and *Pde3b* (Figure 6c). Surprisingly, we find a reduced expression of *Pde3b* in Cas9 and ctrl cells. To dissect if the reduced adipogenesis might be caused by reduced responsiveness of preadipocytes to insulin, we monitored insulin receptor (*IR*) expression which was comparable to wt cells (Figure 6c).

As adipocyte function regarding adiponectin secretion, lipolysis, and insulin-stimulated glucose uptake were performed in fully differentiated 3T3-L1 adipocytes, we also evaluated possible changes in gene expression by qPCR in mature cells of each cell line (Figure 6d-f). Because isoprenaline and forskolin showed no or lower effects on adiponectin secretion and lipolysis, we again monitored expression of β-adrenergic receptors and adenylyl cyclases. As expected, *Adbr3* expression increased during differentiation (Figure 6a, d) and showed a significant reduction in Cas9 and ctrl cells. Interestingly, also *Adbr1* expression increased over differentiation and its expression was significantly reduced in Cas9 and ctrl cells (Figure 6d). Similar to expression in preadipocytes, we did not find any differences in adenylyl cyclase expression (Figure 6e) but again reduced *Pde3b* expression in Cas9 and ctrl cells (Figure 6f). As not only cAMP signalling cascades but also insulin responsiveness was reduced in Cas9 cells, we analysed insulin receptor expression, which was significantly reduced (Figure 6f).

In addition to decreased responsiveness to isoprenaline, forskolin, and insulin, Cas9 and ctrl cells also displayed reduced basal lipolysis. We, therefore, speculated that expression of lipases might additionally account for this effect and analysed adipose triglyceride lipase (*Atgl)*, hormone sensitive lipase (*Hsl)*, and monoacylglycerol lipase (*Mgl*) expression. Expression of all three enzymes increased from preadipocytes to mature adipocytes and we found a trend towards *Hsl* expression reduction in Cas9 and ctrl cells (Suppl. Figure S2).

### Evaluating Cas9 transient expression in 3T3-L1 cells

**Figure 7.**
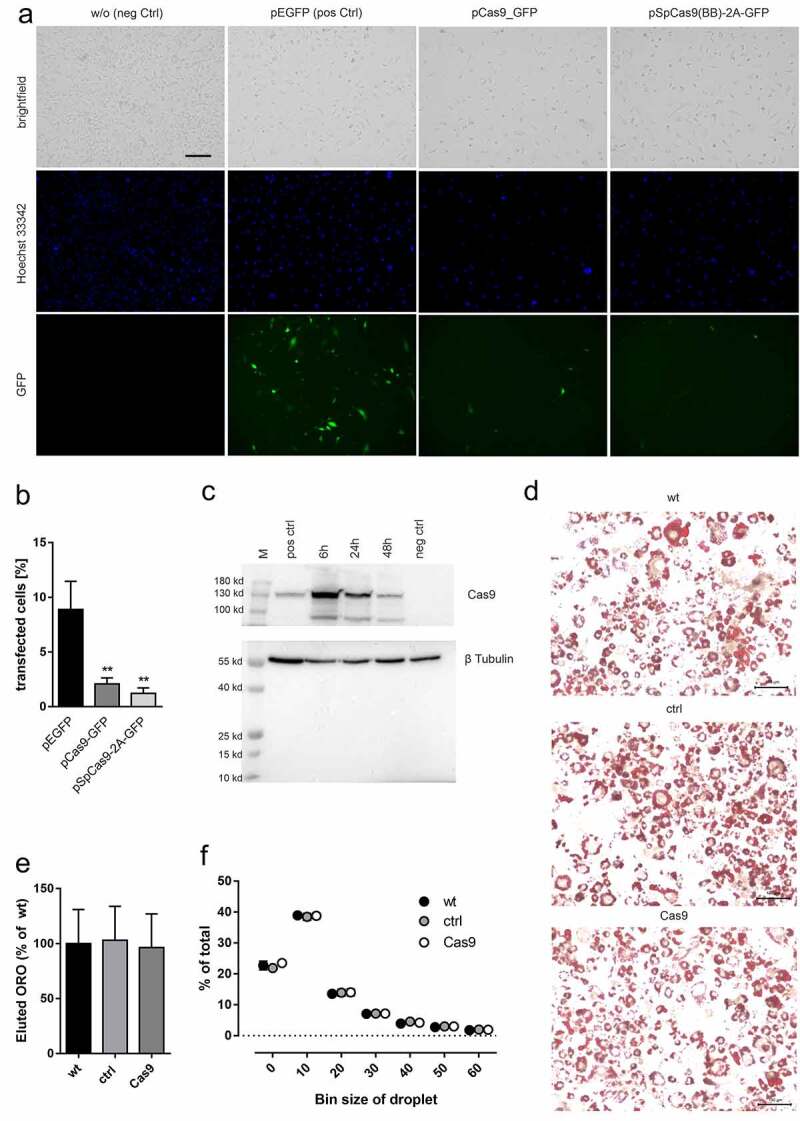
Stable and transient overexpression of Cas9 in 3T3-L1 preadipocytes. (a) 3T3-L1 wt were transfected with pEGFP, pCas9_GFP, and pSpCas9(BB)-2A-GFP and microscopic analysis performed to evaluate transfection efficacy. (b) Quantification revealed in general a low transfection rate which was significantly reduced in cells transfected with plasmids coding for Cas9. Statistical significance was identified using one-way ANOVA (n = 4; ** p < 0.01). (c) Cas9 protein electroporation in 3T3-L1 wt was analysed by Western blot 6 h, 24 h and 48 h post transfection and showed successful Cas9 protein transfection. pos. ctrl: Cas9 OE, neg. ctrl: wt. (d) Cas9 protein transfected 3T3-L1 were differentiated for 10 days and stained with ORO to analyse adipogenesis. No differences were observed comparing Cas9-, ctrl-transfected or wt cells. (e) Total lipid accumulation of Cas9 protein-transfected, control and wt cells was measured by eluted ORO in mature adipocytes (n = 3) with no determinable differences. (f) In mature adipocytes the number of lipid droplets was determined in a defined area (0.2664 mm2) (n = 3) and did not reveal any changes due to transfection of Cas9 protein

The above results hint towards a significant change of 3T3-L1 physiology in cells derived from the cell line constitutively expressing Cas9, yielding cells with lower adipogenesis capacity, responsiveness to cAMP signalling, and insulin sensitivity. In order to investigate if this impairment is caused by the constitutive occurrence of Cas9 in general, we transfected 3T3-L1 cells using a puromycin-selectable plasmid; however, no Cas9 protein was detectable (Suppl. Figure S3). Previous studies have shown that transient expression of Cas9 is sufficient to realize the gene-editing process as its frequency reaches a plateau after 24 h [29]. However, using plasmids for GFP-detectable transient Cas9-expression, we found no expression of Cas9 most likely due to low transfection efficiency (Figure 7a, b). In another approach, we directly transfected cells with the Cas9 protein, which then resulted in measurable amounts of protein for up to 48 h post transfection with highest levels after 6 h and a subsequent decline (Figure 7c). This is well within the functional time window of Crispr/Cas gene knockout. Interestingly, transient expression of Cas9 does not impair 3T3-L1 differentiation as lipid droplet size and ORO staining show no significant differences (Figure 7d-f) indicating that short-term Cas9 expression does not interfere with 3T3-L1 adipogenesis.

## Discussion

We have previously shown that the adhesion receptors GPR64 and GPR126 play a role in adipocyte differentiation and function [22]. In that study, we used a siRNA-mediated approach to reduce receptor amount. As it is already known for this method, the receptor KD was neither fully efficient nor stable until the final days of differentiation. To overcome these drawbacks, we used a genetic KO approach. Therefore, this study aimed to generate a CRISPR/Cas-mediated genetic KO for *Gpr64* and *Gpr126* to analyse the impact of a complete loss-of-function for both receptors. CRISPR-based KO in 3T3-L1 cells was performed in previous studies either by co-transfecting Cas9 plasmid and guide RNA [30–32] or by generating a stably Cas9-expressing cell line followed by guide RNA transfection [10,11]. A drawback of the first approach is that Cas9 expression is not comparable between isolated single clones leading to a lower consistency, as it has been shown that knock-out efficacy depends on Cas9 protein concentration within a cell [33]. Establishing 3T3-L1 cells stably expressing Cas9, however, was not reported to lead to any major impairment in these newly designed cell lines regarding differentiation, mRNA expression, and hormone secretion [10,11] indicating that Cas9 expression would not interfere with 3T3-L1 function. Currently, there are several different cell lines available constitutively expressing Cas9 from various providers including modified 3T3-L1. We obtained one of them to generate one KO clone of each receptor by inducing indels in the very N-terminal part of either *Gpr64* or *Gpr126* leading to premature stop codons, which was confirmed by sequencing (Figure 1, Suppl. Figure S1). Furthermore, we established a control cell line to monitor transfection-based changes.

In a first step, we differentiated the KO cell lines into 3T3-L1 adipocytes to study the effect of *Gpr64* and *Gpr126* KO in this process. We observed majorly impaired differentiation of *Gpr126* KO and a reduced effect of *Gpr64* KO onto adipogenesis (Figure 2). These observations are in line with the previous siRNA-based receptor KD experiments [22]. In further analysis of adipocyte function (adiponectin secretion, lipolysis, insulin-stimulated glucose-uptake, see Figure 3) no response to isoprenaline, an agonist of β-adrenergic receptors with well-characterized properties in adipocytes and 3T3-L1 cells [28,34,35], was observed in all three cell lines. Additionally, the expected effects of forskolin on adiponectin secretion and lipolysis were not observed even in the control cell line.

To rule out the possibility that transfection of the gRNA itself had an effect on adipocyte vitality and gene expression, as described in other cell lines [36,37], we investigated the properties of the original Cas9 overexpressing cell line in comparison to 3T3-L1 wt cells and the control cell line. Compared to wt cells impaired adipogenesis was already detectable in Cas9 cells shown by a diminished amount of accumulated lipids (Figure 4a, b). This reduction is manifested in a lowered number of lipid droplets and a shift towards the smallest droplet size (Figure 4c-f). Surprisingly, we did not observe significant changes in *Pparγ* and *Cebpα* expression (Figure 4c-f), as would have been expected from the lack of cAMP-mediated signalling that was prominent in Cas9 and scramble control cells (Figure 5a) since it has been demonstrated that cAMP responsive element-binding protein (CREB) activation induces adipogenesis in 3T3-L1 cells [38]. It appears that the initial lack of cAMP in Cas9 and control cells is not strong enough to influence these master regulators of differentiation. Only the additional KO of the Gs-coupled *Gpr126* lowered cAMP levels sufficiently to reduce expression of *Pparγ* and *Cebpα* (Figure 2e, f). Thus, the observed significantly lower ORO staining in fully differentiated Cas9 and control cells most probably is the result of impaired lipid accumulation. A major source for accumulated lipids in adipocytes is extracellular glucose, whose uptake is regulated by insulin signalling. Indeed, this insulin-mediated function was impaired in both cell lines (Figure 5d), which can be explained by the reduced expression of the insulin receptor (Figure 6f).

In addition to diminished adipogenesis, likely through the reduced cAMP levels in undifferentiated Cas9 and control cells, we also found a significant impairment of cAMP-mediated processes in differentiated adipocytes such as adiponectin secretion or lipolysis. While wt 3T3-L1 cells displayed the expected decrease in adiponectin secretion and increase in lipolysis upon treatment with isoprenaline or forskolin [28,34,35], Cas9 and control cells failed to adequately respond to both stimuli. This demonstrates that also changes connected to cAMP-signalling pathways are not a consequence of the gRNA transfection process but are instead caused by Cas9 overexpression.

To elucidate the cause of the reduced receptiveness of Cas9 and ctrl cells in response to stimulators of cAMP formation, we performed qPCR analysis monitoring the expression of genes involved in the affected pathways (Figure 6). First, we analysed β-adrenergic receptor expression (Figure 6a, d) as these are expressed in 3T3-L1 cells [34] and necessary to transmit isoprenaline signals [39]. We monitored expression of all three β-adrenergic receptors in preadipocytes and mature adipocytes, where expression of the adipocyte-specific *Adbr3* was increased during differentiation as expected [40]. A significant reduction of this gene was observed in mature adipocytes but not preadipocytes. Interestingly, *Adbr1* expression was also increased in mature adipocytes compared to undifferentiated cells. However, expression in Cas9 cells was significantly decreased in both, preadipocytes and mature adipocytes, when compared to wt cells. This indicates a contribution of *Adrb1* to the loss of isoprenaline stimulability. In addition to impaired isoprenaline responsiveness, we found reduced basal cAMP levels as well as diminished forskolin response in Cas9-derived cells. The analysis of adenylyl cyclase 3, 6, and 7 expression did not reveal differences in comparison to wt cells (Figure 6b, e). Unfortunately, we were not able to design specific primers for *Adcy4, Adcy5*, and *Adcy9*, which are also expressed in adipose tissue [41], as either unintended targets were amplified or the doubling efficiency per qPCR cycle was low. However, since *Adcy3* has been described as the major adenylyl cyclase mediating cAMP production in adipocytes [42], we assume that other isoforms might only have a minor contribution to the observed differences. Cyclic AMP levels are also controlled by degradation through phosphodiesterases. In adipocytes, *Pde3b* is the main isoform expressed [43,44] and, unexpectedly, we found decreased expression in preadipocytes as well as adipocytes (Figure 6c, f). However, it has been previously shown in pulmonary smooth muscle cells that an increase in cAMP can result in increased *Pde3* expression [45]. Therefore, it seems conceivable that the observed reduced amount of cAMP negatively regulates *Pde3* transcript levels in Cas9 adipocytes.

PDE3B is also strongly connected to insulin-signalling and represents a link between insulin and cAMP signalling. Inhibition of PDE3 has been shown to decrease insulin-induced glucose uptake [43], and, therefore, lower amounts of *Pde3b* transcript levels may add to the effect of reduced insulin receptor expression (Figure 6f). Indeed, low *Pde3b* expression can also be a cause for development of insulin insensitivity [46].

Regarding the observed changes, the usefulness of KO clones derived from this cell line is in general questionable, while analysis of GPCR function connected to cAMP-signalling pathways is not feasible at all.

In general, the implementation of gene editing approaches in 3T3-L1 cells is desirable considering the instability and often insufficiency of siRNA-mediated knockdown. Thus, we tested several alternate methods to establish constitutive and transient Cas9 expression. In our hands only the transient transfection of Cas9 protein resulted in considerable amounts of this enzyme in the cells (Figure 7c). There are published studies describing the use of transient and stably Cas9 plasmid transfected cell lines, indicating that Cas9 expression can be achieved through this method. However, in many cases the knockout effects were only compared to an undefined control cell [10,47,48]. Based on our results, it is essential to control specific gene-targeting effects to at least a cell line transfected with Cas9 and scrambled guide RNA, while it would be advisable to simultaneously monitor the effects of Cas9 expression in comparison to untreated wt 3T3-L1 cells.

In sum, we found vast impact of permanent Cas9 overexpression onto the functionality of 3T3-L1 cells. In the cell line used in this study, *S. pyogenes* Cas9 is constitutively expressed which appears to induce significant changes in expression levels of several genes necessary for adipogenesis and adipocyte function. Similar findings have been observed in neuronal cells constitutively expressing Cas9, where RNAseq analysis revealed 400 genes to be differentially expressed due to prolonged Cas9 expression [49]. As it is not described how and where Cas9 is integrated into the genome of 3T3-L1 cells, alterations on the genomic level may be the underlying cause of the observed changes. As the transient expression of Cas9 protein did not interfere with 3T3-L1 differentiation (Figure 7d-f) it represents a suitable alternative to chromosomal targeting of 3T3-L1 cells.

## Materials and methods

### Materials

If not stated otherwise standard chemicals were obtained from Sigma-Aldrich (Munich, Germany) or C. Roth GmbH + Co. KG (Karlsruhe, Germany).

### Cell culture and differentiation

3T3-L1 wt cells were obtained from ATCC (LGC Standards, Wesel, Germany) and stably Cas9-expressing 3T3-L1 cell line was purchased from PrimCells (San Diego, CA, USA).

All cell lines were cultured in CELLSTAR® Filter Cap Cell Culture Flasks (GBO, Frickenhausen, Germany) in culture medium (DMEM supplemented with 10% FBS, 100 U/ml penicillin and 100 µg/ml streptomycin) at 37°C in a humidified atmosphere containing 5% CO_2_. Cells were maintained until 70–80% confluence and split every 2–3 days. Cell differentiation into mature adipocytes was induced 2 days after reaching confluence by culturing in induction medium (culture medium containing 1 µg/ml insulin, 0.25 µM dexamethasone, 0.5 mM IBMX, 2 µM rosiglitazone). Cells were kept in the induction medium for 3 days before change to differentiation medium (culture medium containing 1 µg/ml insulin) for further 3 days. Subsequently, culture medium was changed every other day until completion of differentiation at day 10 post induction [50].

### Gpr64 and Gpr126 knock-out generation

Generation of *Gpr64* and *Gpr126* knock-out was done in 3T3-L1 cells overexpressing Cas9. Specific gRNA was designed via IDT tools (IDT, Coralville, IA, USA) for *Gpr126* (5ʹ-GGAGACGTAAAGGTACCGGA-3ʹ) and *Gpr64* (5ʹ-AACTGTACAGATTCGGCCAA-3ʹ). Knock-out was induced as previously described [51]. Briefly, gRNA was diluted to 7.5 nM in 100 µl of Opti-MEM and 4.8 µl of RNAiMAX (ThermoFisher Scientific, Darmstadt, Germany) in Opti-MEM to 100 µl. After 5 minutes incubation, both mixtures were combined and transferred to a well of 24-well plate and incubated for 20 minutes. Afterwards, 10,000 3T3-L1 Cas9 cells were added per well. Edited cells were sorted to single clones by FACS and propagated in culture medium. Cells were harvested and DNA was prepared for Illumina library [51]. Libraries were sequenced on MiSeq (Illumina) and analysed by CRISPResso [52], aiming for an insertion or deletion (indel) causing a frameshift to a premature stop codon. Results from next generation sequencing investigations confirmed the knock-out and are shown in Suppl. Figure S1. The 3T3-L1 scramble control cell line was generated equivalently by using a gRNA with a scrambled sequence without any alignment to the mouse genome (OriGene Technologies, Inc, Rockville, USA).

### RNA extraction and real-time quantitative-PCR

RNA isolation was performed using the ReliaPrep™ RNA Miniprep System (Promega, Mannheim, Germany) according to the manufactures’ instructions. cDNA was synthetized by SuperScript II™ Reverse Transcriptase (ThermoFisher Scientific). Quantitative PCR was performed using Platinum® SYBR-Green qPCR SuperMix-UDG (ThermoFisher Scientific), 10 ng cDNA, 1.2 µM primer mix (primer sequences are given in the supplements) at the CFX Connect™ Real-Time PCR Detection System (Bio-Rad Laboratories GmbH, Feldkirchen, Germany). Obtained Ct values were normalized to *Actb2*. All primers were synthesized by Microsynth Seqlab (Göttingen, Germany).

### Gpr64 cell surface expression

Cell surface expression of endogenously expressed GPR64 in ctrl and 64ko cells was determined in 3T3-L1 preadipocytes and on day 4 of differentiation using an indirect ELISA. Cells were fixed with 4% formaldehyde for 20 min at room temperature before blocking for 1 h at 37°C. Then, cells were incubated with the primary anti-Gpr64 antibody (1:100, AF7977, R&D, Minneapolis, MN, USA) for 1 h at room temperature. Afterwards, the secondary POD-labelled antibody (1:500, HAF016, R&D) was added equivalently. To determine background, incubation only with secondary antibody was performed. Blocking and antibody dilution was done in media supplemented with 10% FBS. Cell surface expression was analysed using *o*-phenylenediamine solved in substrate buffer (0.1 M citric acid, 0.1 M Na_2_HPO_4_) containing 0.2% H_2_O_2_. The reaction was stopped after 20–40 min with 1 M HCl containing Na_2_SO_3_. OD readings were recorded at 492 nm using the Sunrise microplate reader (Tecan, Männedorf, Switzerland) and normalized by detracting background readings.

### cAMP-assay

Cyclic AMP accumulation was performed in confluent, undifferentiated 3T3-L1 cells. Cells were washed with serum- and phenol red-free DMEM containing 1 mM IBMX and incubated with the indicated stimuli for 15 min. Subsequently, cells were harvested in LI buffer (5 mM HEPES, 0.3% Tween-20, 0.1% BSA, and 0.5 mM IBMX) and frozen at -80°C overnight. The AlphaScreen^TM^ cAMP functional assay (PerkinElmer, Rodgau, Germany) was used to determine the amount of cAMP according to the manufacturer’s protocol using the EnVision 2105 Multimode Plate Reader (PerkinElmer).

### Electroporation

3T3-L1 preadipocytes were transfected using an electroporation system (Neon Transfection System, Invitrogen) with either 5 µg Plasmid/10^6^ cells or with RNP complex according to manufacturer’s instructions (IDT). Cells transfected with a plasmid coding a selection marker were incubated with 1 ug/ml puromycin 24 h after transfection (Suppl. Figure S3A). The following plasmids and proteins were used: pEGFP-C1 (Clonetech Palo-Alto, CA, USA), pCas9_GFP (Addgene, #44,719 [53],), pSpCas9(BB)-2A-GFP (Addgene, #48,138 [54],), pSpCas9(BB)-2A-Puro (Addgene, #62,988 [54],), Alt-R® S.p. HiFi Cas9 Nuclease V3 (IDT, #1,081,061).

### Cell count and confluence analysis

Cell confluence was determined in 3T3-L1 preadipocytes 24 h, 48 h, 72 h and 96 h after puromycin exposure. Transfection efficiency was analysed in 3T3-L1 preadipocytes 24 h after electroporation. Total cell count was determined in (pre-)adipocytes using Hoechst 33342. Cells were stained for 30 min at 37°C with Hoechst 33342 (1 μg/ml, Sigma-Aldrich). Images were taken and analysed using the Celigo Image Cytometer (Nexcelom Bioscience, Lawrence, MA, USA).

### Lipolysis

Lipolysis rate in mature 3T3-L1 cells was analysed by free glycerol in supernatant. After 5 h starvation in low glucose (5.6 mM) DMEM containing 1% FBS, cells were incubated for 16 h with indicated substances. For determination, 20 µl of supernatant were mixed with 150 µl of Free Glycerol Reagent (Sigma-Aldrich) and incubated for 10 min at 37°C. OD readings were recorded at 540 nm using the Sunrise microplate reader (Tecan) and the free glycerol amount was normalized to total cell count.

### Adiponectin secretion

To compare adiponectin secretion, mature 3T3-L1 cells were starved in serum- and phenol red-free media overnight before stimulation for 24 h. Secreted adiponectin was measured in supernatant using a mouse adiponectin ELISA kit (ThermoFisher Scientific) following the manufacturer’s instructions and normalized to total cell count.

### Glucose uptake assay

Glucose uptake of 3T3-L1 adipocytes was analysed in 12-well plates. After starvation overnight in serum-free, low glucose (5.6 mM) media the indicated substances were added for 1 h. For induction of glucose uptake, cells were simulated with 100 nM insulin for 15 min. Following, 0.5 µCi/ml [3 H]-deoxy-D-glucose and 100 µM 2-deoxy-glucose were added for 30 min. The cells were washed with ice-cold PBS twice, before lysis in RIPA buffer (150 mM NaCl, 1% (v/v) Triton X-100, 0.5% (w/v) Na-deoxycholate, 0.1% (w/v) SDS, 50 mM Tris (pH8.0)). Accumulated [^3^H]-deoxy-D-glucose was measured using a scintillation counter (PerkinElmer). Measurement was normalized to protein amount analysed by using the Pierce BCA kit (ThermoFisher Scientific).

### Adipocyte staining and droplet analysis

Differentiated 3T3-L1 cells were fixed in 4% formaldehyde for 1 h at room temperature. Fixed cells were washed with 60% isopropanol, dried and stained with Oil Red O (ORO) solution in 60% isopropanol at concentration of 2.1 g/l. Staining was performed for 10 minutes and cells were subsequently washed four times with dH_2_O. Pictures of stained cells were acquired by Leica LAS EZ software (Leica Microsystems GmbH, Wetzlar, Germany). ORO bound in cell lipids was eluted by 50 µl of 100% isopropanol and OD was measured at 500 nm using the Sunrise™ photometer (Tecan). Acquired pictures were subjected to lipid droplet analysis as described before [22,55] using ImageJ.

### Western blot analysis

3T3-L1 cells were harvested in RIPA buffer (Sigma-Aldrich) containing protease- and phosphatase inhibitors (ThermoFisher Scientific), incubated at 4°C for 60 min before centrifugation (16,000 rpm, 4°C, 15 min). Supernatant-containing total protein was analysed using bicinchoninic acid (BCA) assay according to manufacturer’s indications (ThermoFisher Scientific). 20 µg total protein were used for SDS PAGE and transferred to a 0.45 µm PVDF membrane using tank blotting (60 min, 100 V, 4°C). Membranes were blocked with 5% milk powder in TBST for 1 h at room temperature before primary antibody in 5% BSA in TBST was added at 4°C overnight. Secondary antibody in TBST was added for 60 min at room temperature. Chemiluminescence signals were detected using a gel documentation system. Following antibodies were used: anti-Cas9 (CST, #19,526, 1:1000), anti-beta-Tubulin (CST, #2146, 1:1000), and anti-rabbit-HRP (CST, #7074, 1:1000). Marker for size determination was obtained from ThermoFisher Scientific (PageRuler^TM^ Prestained Protein ladder).

### Statistical analysis

Statistical significance was determined using two-tailed Student’s *t*-test for comparison of two groups. More than two groups were compared by one-way ANOVA using GraphPad Prism 8.4 software. When analysing adipocyte function, significance was evaluated by paired one-way ANOVA as adipocyte differentiation can vary in different batches. P-values below 0.05 were considered to be significant.

## Supplementary Material

Supplemental Material

## Data Availability

The data that support the findings of this study are openly available in ‘zenodo’ at https://doi.org/10.5281/zenodo.5543597.
