# Supplementary material for: Evaluating the feasibility of Cas9 overexpression in 3T3-L1 cells for generation of genetic knock-out adipocyte cell lines
Source: Adipocyte. 2021 Dec 16;10(1):631–45. doi: 10.1080/21623945.2021.1990480 (PMC8735834; doi:10.1080/21623945.2021.1990480)
Supplement: Supplemental Material [file KADI_A_1990480_SM9221.zip › Suchy_Kaczmarek_et_al_Suppl_revision_proof.docx]

**Supplements**

**Evaluating the feasibility of Cas9 overexpression in 3T3-L1 cells for generation of genetic knock-out adipocyte cell lines**

**AUTHORS**

Tomás Suchý^1†^, Isabell Kaczmarek^1†^, Tomislav Maricic^2^, Christian Zieschang^1^, Torsten Schöneberg^1^, Doreen Thor^1#^*, Ines Liebscher^1#^*.

**AFFILIATIONS**

^1^Rudolf Schönheimer Institute of Biochemistry, Medical Faculty, Leipzig University, Johannisallee 30, 04103 Leipzig, Germany

^2^Department of Evolutionary Genetics, Max-Planck-Institute for Evolutionary Anthropology, Deutscher Platz 6, 04103, Leipzig, Germany

^†#^ These authors contributed equally to this work.

**CONTACT INFORMATION**

^*^To whom correspondence should be addressed:

ines.liebscher@medizin.uni-leipzig.de; +49 341 9722 141

doreen.thor@medizin.uni-leipzig.de; +49 341 9722 177

### Supplements


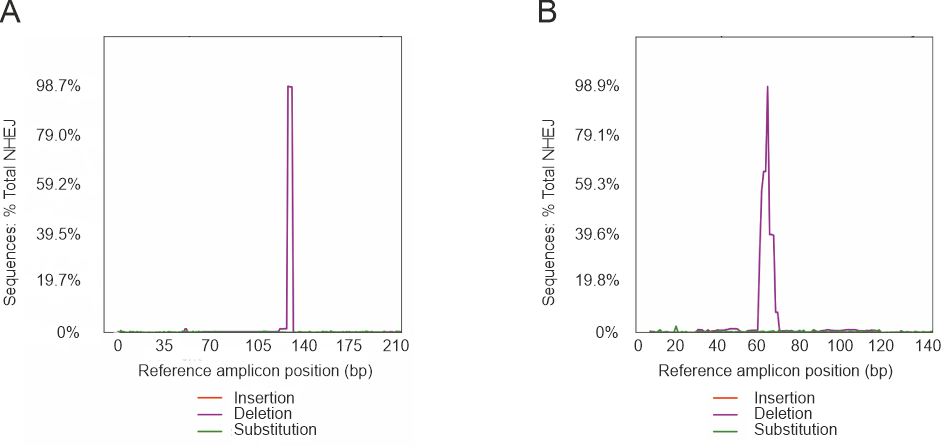


**Suppl. Figure S1. Generation of *Gpr64* and *Gpr126* knock-out cell lines.** 3T3-L1 cells carrying *Cas9* gene were transfected with gRNA targeting *Gpr126* or *Gpr64*, respectively*.* (**A**) The majority of sequences (98.8%, n = 1436) derived from the selected clone showed an ORF-shifting deletion in *Gpr126* gene. Specifically, a 4-nucleotide deletion was observed in position 76668-76671 (NCBI RefSeq: NC_000076.6). **(B)** *Gpr64* editing resulted in four major altered sequences in 27%-23%-20%-6% ratio. In total, 98.9% of sequences showed an ORF-shifting deletion (n = 5618). Specifically, the edit resulted in 1 – 8 – 4 – 8 bp deletions in position 160474787 – 160474797 (NC_000086.7).


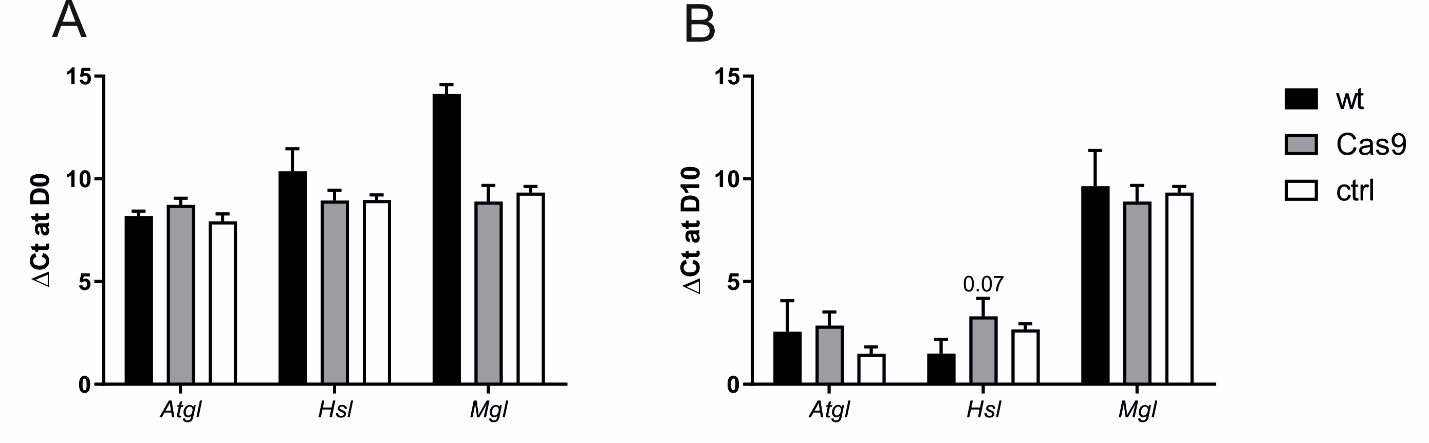


**Suppl. Figure S2. Analysis of mRNA expression in preadipocytes (A) and mature adipocytes (B) for genes involved in lipolysis.** Due to impaired lipolysis in mature adipocytes of Cas9 and ctrl cells expression of lipases was investigated (n=4-7). Ct values were normalized to *Actb2* (Ct=15.28±0.245). Data is shown as mean ± SEM. Statistical significance was identified by one-way ANOVA.


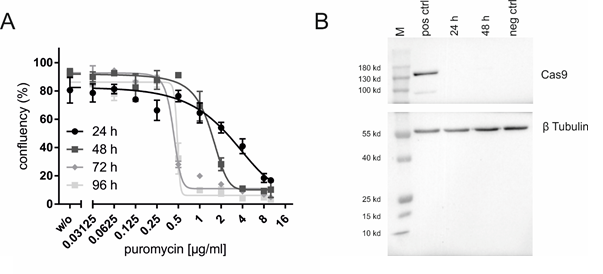


**Suppl. Figure S3. Analysis of 3T3-L1 transfection with puromycin-selectable Cas9-coding plasmid. (A)** Confluent 3T3-L1 were incubated with various concentrations of puromycin and showed a time and concentration dependent decline of survival. Data is shown as mean ± SEM, n = 3. **(B)** 3T3-L1 wt cells were transfected with pSpCas9(BB)-2A-Puro and incubated for 24 h or 48 h in selection media containing puromycin (1 µg/ml) Following, western blot analysis could not show Cas9 expression (pos. ctrl: Cas9 OE, 24 h / 48 h: puromycin selection for 24 h or 48 h, respectively, neg. ctrl: 3T3-L1 wt cells).

**Suppl. Table S1.** Primer sequences used for reverse transcription and qPCR.

| Gene | Primer sense (5‘->3‘) | Primer antisense (5‘->3‘) |
| --- | --- | --- |
| *Actb2* | GCTCTTTTCCAGCCTTCCTT | CGGATGTCAACGTCACACTT |
| *Adcy6* | TGCGTGAGGTAACAGGTGTGAA | TCAAACTGCCATTTCCGTAGGC |
| *Adcy7* | AACCCTTCTGTGGGTCAAGGA | CCACTCGGAGCATTGACCTGT |
| *Adcy8* | TTGCTCTGCTTGCCTGAAGAC | CGATGTTGTCGAAAGGCAGCTC |
| *Adrb1* | ACACTGGGCATCATCATGGG | AGAAGACGAAGAGGCGATCC |
| *Adrb2* | TCGAGCGACTACAAACCGTC | CCAGAACTCGCACCAGAAGT |
| *Adrb3* | CCACCGCTCAACAGGTTTGAT | GGGGCAACCAGTCAAGAAGAT |
| *Atgl* | CCACTGTCTTGCGCCACCTA | TGACGCTGGCATTCTTCCCA |
| *Gpr126* | TGGAGCATCTCAACCCAAGC | ACATTATTCCGTCTTGACAGAGAGT |
| *Gpr64* | CACTAACTCCACCACACTCCT | GAAACCCCATTCCTCTCGGT |
| *Hsl* | CATCAACCGACCAGGAGTGCT | GCAGCCTTTGTGTAGCGTGA |
| *IR* | GGAATGTGGGGATGTCTGTCC | CTGTGCAGCCATGTGACTTA |
| *Mgl* | GCCCTCATCTTTGTGTCCCAT | GCAAATACCAGCATGTCCAGCC |
| *Pde3b* | CCTGGGCTTGGACCACTTCTT | TTTCTCCCAGCGACACGCA |
| oligo-dt | TTTTTTTTTTTTTTTTTTTTTTTVN |  |
| random hex | NNNNNN |  |
