# Supplementary material for: Evaluating the feasibility of Cas9 overexpression in 3T3-L1 cells for generation of genetic knock-out adipocyte cell lines
Source: Adipocyte. 2021 Dec 16;10(1):631–45. doi: 10.1080/21623945.2021.1990480 (PMC8735834; doi:10.1080/21623945.2021.1990480)
Supplement: Supplemental Material [file KADI_A_1990480_SM9221.zip › Suchy_Kaczmarek_et_al_Suppl_revision_proof.docx.pdf]

Supplements

Evaluating the feasibility of Cas9 overexpression in 3T3-L1 cells for generation of genetic knock-out adipocyte cell lines

AUTHORS

Tomás Suchý[1†], Isabell Kaczmarek[1†], Tomislav Maricic[2], Christian Zieschang[1], Torsten Schöneberg[1], Doreen Thor[1#*], Ines Liebscher[1#*].

AFFILIATIONS

[1]Rudolf Schönheimer Institute of Biochemistry, Medical Faculty, Leipzig University, Johannisallee 30, 04103 Leipzig, Germany

[2]Department of Evolutionary Genetics, Max-Planck-Institute for Evolutionary Anthropology, Deutscher Platz 6, 04103, Leipzig, Germany

[†#] These authors contributed equally to this work.

CONTACT INFORMATION

[*]To whom correspondence should be addressed:

ines.liebscher@medizin.uni-leipzig.de; +49 341 9722 141

doreen.thor@medizin.uni-leipzig.de; +49 341 9722 177

Supplements

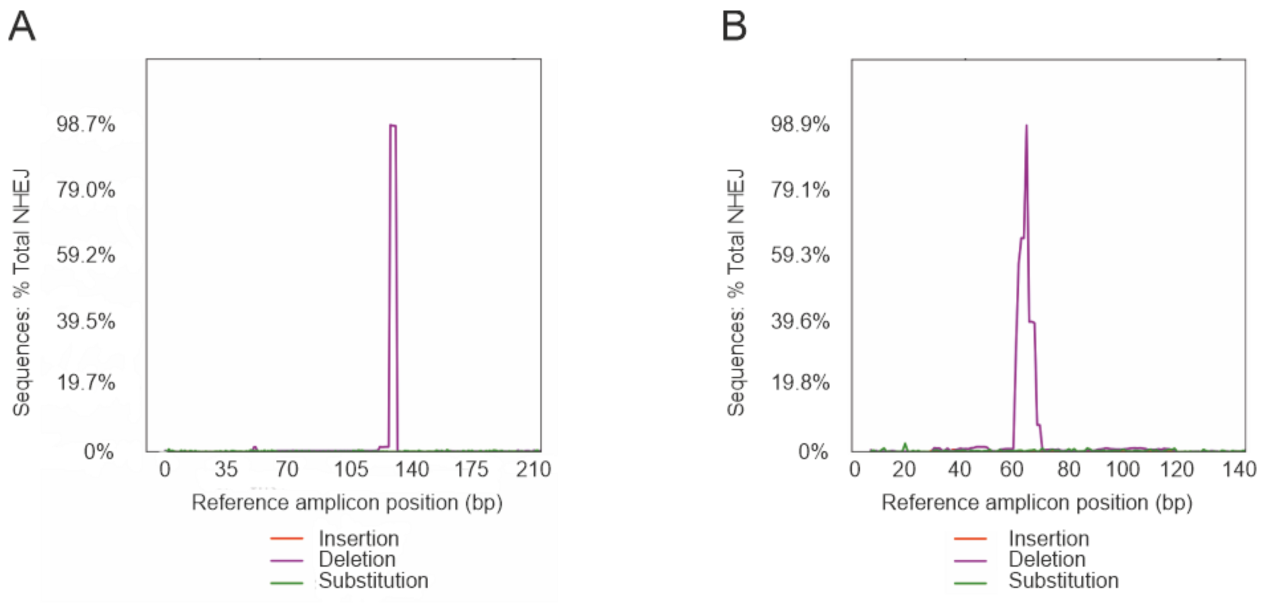

Suppl. Figure S1. Generation of *Gpr64* and *Gpr126* knock-out cell lines. 3T3-L1 cells carrying *Cas9* gene were transfected with gRNA targeting *Gpr126* or *Gpr64*, respectively. (**A**) The majority of sequences (98.8%, n = 1436) derived from the selected clone showed an ORF-shifting deletion in *Gpr126* gene. Specifically, a 4-nucleotide deletion was observed in position 76668-76671 (NCBI RefSeq: NC_000076.6). **(B)** *Gpr64* editing resulted in four major altered sequences in 27%-23%-20%-6% ratio. In total, 98.9% of sequences showed an ORF-shifting deletion (n = 5618). Specifically, the edit resulted in 1 – 8 – 4 – 8 bp deletions in position 160474787 – 160474797 (NC_000086.7).

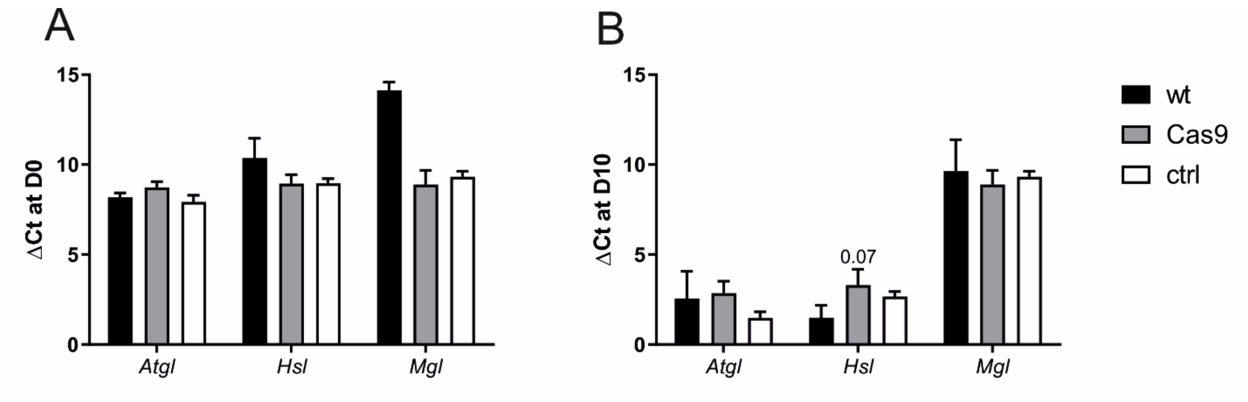

Suppl. Figure S2. Analysis of mRNA expression in preadipocytes (A) and mature adipocytes (B) for genes involved in lipolysis. Due to impaired lipolysis in mature adipocytes of Cas9 and ctrl cells expression of lipases was investigated (n=4-7). Ct values were normalized to *Actb2* (Ct=15.28±0.245). Data is shown as mean ± SEM. Statistical significance was identified by one-way ANOVA.

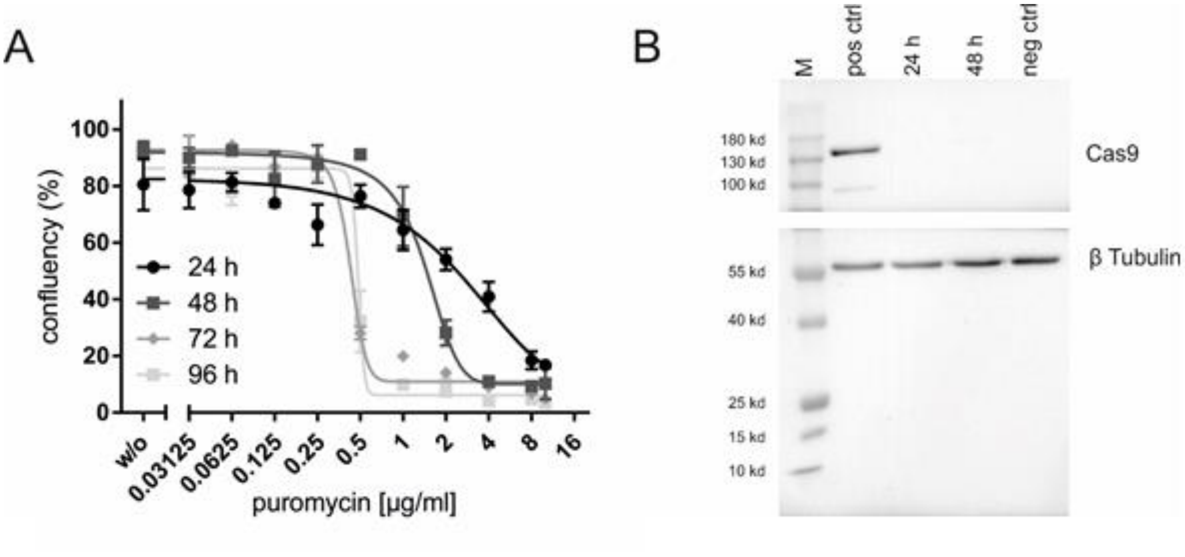

Suppl. Figure S3. Analysis of 3T3-L1 transfection with puromycin-selectable Cas9-coding plasmid. (A) Confluent 3T3-L1 were incubated with various concentrations of puromycin and showed a time and concentration dependent decline of survival. Data is shown as mean ± SEM, n = 3. **(B)** 3T3-L1 wt cells were transfected with pSpCas9(BB)-2A-Puro and incubated for 24 h or 48 h in selection media containing puromycin (1 µg/ml) Following, western blot analysis could not show Cas9 expression (pos. ctrl: Cas9 OE, 24 h / 48 h: puromycin selection for 24 h or 48 h, respectively, neg. ctrl: 3T3-L1 wt cells).

Suppl. Table S1. Primer sequences used for reverse transcription and qPCR.

Gene	Primer sense (5‘->3‘)	Primer antisense (5‘->3‘)
Actb2	GCTCTTTTCCAGCCTTCCTT	CGGATGTCAACGTCACACTT
Adcy6	TGCGTGAGGTAACAGGTGTGAA	TCAAACTGCCATTTCCGTAGGC
Adcy7	AACCCTTCTGTGGGTCAAGGA	CCACTCGGAGCATTGACCTGT
Adcy8	TTGCTCTGCTTGCCTGAAGAC	CGATGTTGTCGAAAGGCAGCTC
Adrb1	ACACTGGGCATCATCATGGG	AGAAGACGAAGAGGCGATCC
Adrb2	TCGAGCGACTACAAACCGTC	CCAGAACTCGCACCAGAAGT
Adrb3	CCACCGCTCAACAGGTTTGAT	GGGGCAACCAGTCAAGAAGAT
Atgl	CCACTGTCTTGCGCCACCTA	TGACGCTGGCATTCTTCCCA
Gpr126	TGGAGCATCTCAACCCAAGC	ACATTATTCCGTCTTGACAGAGAGT
Gpr64	CACTAACTCCACCACACTCCT	GAAACCCCATTCCTCTCGGT
Hsl	CATCAACCGACCAGGAGTGCT	GCAGCCTTTGTGTAGCGTGA
IR	GGAATGTGGGGATGTCTGTCC	CTGTGCAGCCATGTGACTTA
Mgl	GCCCTCATCTTTGTGTCCCAT	GCAAATACCAGCATGTCCAGCC
Pde3b	CCTGGGCTTGGACCACTTCTT	TTTCTCCCAGCGACACGCA
oligo-dt	TTTTTTTTTTTTTTTTTTTTTTTVN	
random hex	NNNNNN	
